# Pretreatment with Resveratrol Prevents Neuronal Injury and Cognitive Deficits Induced by Perinatal Hypoxia-Ischemia in Rats

**DOI:** 10.1371/journal.pone.0142424

**Published:** 2015-11-06

**Authors:** Olatz Arteaga, Miren Revuelta, Leyre Urigüen, Antonia Álvarez, Haizea Montalvo, Enrique Hilario

**Affiliations:** 1 Department of Cell Biology & Histology, School of Medicine & Dentistry, University of the Basque Country (UPV/EHU), Leioa, Bizkaia, Spain; 2 Department of Pharmacology, School of Medicine & Dentistry, University of the Basque Country (UPV/EHU), Leioa, Bizkaia, Spain; 3 Centro de Investigación Biomédica en Red de Salud Mental (CIBERSAM), Madrid, Spain; Albany Medical College, UNITED STATES

## Abstract

Despite advances in neonatal care, hypoxic-ischemic brain injury is still a serious clinical problem, which is responsible for many cases of perinatal mortality, cerebral palsy, motor impairment and cognitive deficits. Resveratrol, a natural polyphenol with important anti-oxidant and anti-inflammatory properties, is present in grapevines, peanuts and pomegranates. The aim of the present work was to evaluate the possible neuroprotective effect of resveratrol when administered before or immediately after a hypoxic-ischemic brain event in neonatal rats by analyzing brain damage, the mitochondrial status and long-term cognitive impairment. Our results indicate that pretreatment with resveratrol protects against brain damage, reducing infarct volume, preserving myelination and minimizing the astroglial reactive response. Moreover its neuroprotective effect was found to be long lasting, as behavioral outcomes were significantly improved at adulthood. We speculate that one of the mechanisms for this neuroprotection may be related to the maintenance of the mitochondrial inner membrane integrity and potential, and to the reduction of reactive oxygen species. Curiously, none of these protective features was observed when resveratrol was administered immediately after hypoxia-ischemia.

## Introduction

Neonatal hypoxia-ischemia and subsequent brain damage still continue to be an alarming socio-sanitary problem, being considered the single-most important cause of acute mortality and chronic disability in newborns worldwide [1–3]. The result of a deprivation of oxygen and glucose to the brain can lead to death or have severe neurological consequences such as cerebral palsy, mental retardation, visual and hearing impairment, learning and behavioral disabilities, attention deficits, hyperactivity and epilepsy [4–7]. The severity of neonatal encephalopathy depends on the intensity, duration and location of the insult [8,9]. Around 15–20% of affected newborns will die in the postnatal period and an additional 25% will develop severe and permanent neuropsychological sequelae [10]. Only a small percentage of infants with severe injury who survive without any handicap [11,12].

Although theoretically all cell types are affected by hypoxia-ischemia, they do not respond in the same manner. The cells most sensitive to oxygen deprivation are neurons, which exhibit selective vulnerability [13,14], whereas astrocytes are more resistant. Nevertheless, hypoxia-ischemia can prejudice the survival of astrocytes and reduce their capacity for excitatory neurotransmitter uptake and free radical scavenging, which ultimately also influences the survival of neurons [15,16]. Oligodendrocytes, the myelinating cells of the central nervous system, are also particularly vulnerable to hypoxia-ischemia. Damage to these cells leads to myelination deficits, white matter lesions and the destruction of gray matter oligodendrocyte progenitors [17,18].

Hypoxia-ischemia associated brain damage due to impaired glucose and oxygen supply results in immediate neuronal injury and the exhaustion of cellular energy stores, which lead to a multi-faceted cascade of biochemical events. Two distinct metabolic phases have been recognized in the ensuing neurological damage: the first is the primary energy failure due to the hypoxic-ischemic event and the second is a consequence of the re-oxygenation which takes place some hours later [19,20]. Oxidative stress, which is defined as an imbalance between oxidant and antioxidant factors, is considered to be a major contributor to ischemic brain injury [21], because it is an important consequence of the neurotransmitter-mediated toxicity which follows perinatal hypoxia-ischemia. All these changes result in mitochondrial dysfunction and the generation of more injurious reactive oxygen species (ROS), which oxidatively damage cell constituents, and ultimately lead to neuronal damage or death [22,23]. The human antioxidant defense system does not accelerate in maturation until the late third trimester of pregnancy and as a consequence the human newborn has a relative deficiency in brain superoxide dismutase and glutathione peroxidase [24]. In this sense, it is reasonable to assume that exogenous antioxidant therapy may help to prevent cellular damage when perinatal asphyxia occurs.

Resveratrol (3,5,4′-trihydroxystilbene) (RVT) is a phytoalexin produced by different plant species, including grapevines, peanuts and pomegranates [25,26]. The most common dietary source of resveratrol is red wine, and it is believed to be an important factor in the French Paradox, a term which refers to the observation that the French population has a very low incidence of cardiovascular disease, in spite of a diet which is high in saturated fats [27,28]. This polyphenol has been found to play a fundamental role in neuroprotection in models of neurodegeneration such as Alzheimer's, Parkinson's or Huntington's disease [29–32], ischemia and brain and spinal cord injury [33–36]. The neuroprotective effects of resveratrol are due to its antioxidant activity associated with its stilbene structure with two phenol rings. These confer it with the ability to scavenge a variety of free radicals, including lipid peroxyl and carbon-centered radicals, and ROS. In addition, it has also been found to be able to upregulate the expression of several antioxidant enzymes [26,32–37].

Thus the aim of the present work was to characterize the effects of resveratrol administered as a preventive agent (before hypoxic-ischemic injury) or as a therapeutic agent (after hypoxia-ischemia injury) on morphological and cellular damage and on behavioral impairments in hypoxic-ischemic neonatal rats. We chose the Rice-Vannucci rat model to provoke hypoxic-ischemic brain injury because it is a perinatal asphyxia model broadly used to investigate cerebral damage and because the maturity of the rat CNS on postnatal day (P7) is similar to that of human term babies [38,39]. To this end, we evaluated brain damage, focusing on the three principal cell populations of the CNS (neurons, astrocytes and oligodendrocytes), which are the key players in the brain response to insults. Moreover, we analyzed mitochondria and ROS production as these are involved in the cascade triggered after hypoxia-ischemia.Finally, we carried out behavioral studies to evaluate the long-term effects of RVT on cognitive function.

## Materials and Methods

### Animals and Ethics Statement

All surgical and experimental procedures were carried out in strict accordance with the recommendations in the Guide for the Care and Use of Laboratory Animals of the National Institutes of Health, as well as in the European Communities directive 2010/63/EU regulating animal research. Experimental protocols were approved by the Committee on the Ethics of Animal Experiments of the University of the Basque Country (UPV/EHU) (Permit Number: CEEA/ 341-344/2014/ALVAREZDIAZ). All surgery was performed under inhaled isoflurane anesthesia, and all efforts were made to minimize suffering.

### Cerebral hypoxia-ischemia damage

Neonatal hypoxia-ischemia was induced using the method originally described for immature rats [40] with slight modifications. Thus, seven day-old (P7) Sprague Dawley rats were anesthetized with inhaled isoflurane (3.5% for induction, 1.5% for maintenance) in oxygen, and the left common carotid artery was isolated from nerve and vein and ligated at two locations with 6–0 surgical silk. The common carotid was then transected between the ligatures to guarantee that blood flow through the ipsilateral carotid circulation was absent throughout the experiment. The wound was sutured and the animals were allowed to recover for 2 h in their cages. Once fully recovered from anesthesia, pups were then placed in a humidified container maintained at 36°C. Hypoxia-ischemia was induced by perfusing the container with humidified 8% oxygen in a nitrogen gas mixture with a flow of 5 L/min for 2 h and 15 minutes. After hypoxic exposure, pups were returned to their biological mothers until they were euthanized with sodium pentobarbital (100 mg/kg) injected intraperitoneally at 0 hours, 3 hours and 12 hours after hypoxic-ischemic insult, on postnatal day 14 (P14) or on postnatal day 90 (P90) for experimental studies. If rats showed signs of increased or decreased respiratory rate, decreased activity, loss of appetite, isolation from littermates (indicative of pain or distress in the animal) they were euthanized by trained personnel of the animal unit of the University of the Basque Country (UPV/EHU) using sodium pentobarbital (in neonates) or carbon dioxide (after weaning). After weaning, animals were housed in individual stalls, and maintained in a climate-controlled environment on a 12-hour light/dark cycle where they had free access to food and water.

## Experimental Groups

Five experimental groups were established for histological evaluation. Control rat pups were randomly chosen and had neither common carotid artery ligation nor a period of hypoxia (Control, n≥5). The hypoxic-ischemic group (HI, n≥8), consisted of animals subjected to hypoxia-ischemia, who received treatment vehicle only [normal saline containing dimethyl sulfoxide (DMSO)]. Resveratrol (Sigma-Aldrich Co. Ltd., Gillingham, UK) was dissolved in DMSO (The Sigma Chemical Co., UK), diluted in normal saline and injected intraperitoneally, as indicated by West et al. (2007) [41]. One group (n≥8) received a single dose of 20 mg/kg resveratrol 10 minutes before hypoxia (RVT-b) and the other group (n≥7), immediately after the hypoxic event (RVT-a). Animals that received treatments were randomly assigned among HI groups. A control+vehicle group was established to test the response to the vehicle, but was not found to be different with respect to the control group.

Since we did not observe any morphological signs of neuroprotection with resveratrol administered after ischemia and in order to avoid unnecessary animal experimentation, we decided not to test the efficacy of post-administered resveratrol in flow cytometry and long-term behavioral studies. Thus, for flow cytometry studies, three experimental groups (control, HI and RVT-b) (n≥5) and three different time points after hypoxic-ischemic insult (0 h, 3 h and 12 h) were evaluated based on the morphological results. Similarly, long-term behavioral studies, that were carried out using P90 control (n = 16), HI (n = 14) and RVT-b (n = 10) rats.

### Histological evaluation

#### Tissue processing

Seven days after surgery (on P14), rats were deeply anesthetized with sodium pentobarbital and perfused intracardially with PBS followed by 4% formaldehyde in 0.1 M PBS (pH 7.2–7.4). Brains were removed and immersed in the same fixative solution at 4°C overnight. After dehydration with graded ethanol and xylene, brains were embedded in paraffin wax and cut in 5 μm coronal sections at interaural distance 5.40 mm and bregma -3.60 mm level, according to the Paxinos and Watson atlas [42]. These sections were stained with Nissl (cresyl violet), or immunolabeled with the antibody to myelin basic protein (MBP), to visualize neurons and oligodendrocytes, respectively.

#### Assessment of brain injury

To quantify the extent of infarction and to assess the severity of tissue injury, 5 μm paraffin embedded sections were stained with cresyl violet (Sigma-Aldrich Co. Ltd., Gillingham, UK). Photographs of the whole brain from different experimental groups were taken with a Carl Zeiss Stemi 2000-C stereomicroscope. The area of infarction was defined as the area which exhibited loss of the normal cresyl violet staining pattern. Image J software (public domain, National Institutes of Health, http://rsbweb.nih.gov/ij/) was used to measure cross-sectional areas from the rhinal sulcus to the interhemispheric fissure of the left (contralateral side) and right hemispheres (ipsilateral side), based on the intensity and uniformity of the staining. Measurements were performed by a researcher who was blind to the conditions of the treatment. The extent of damage was calculated for both hemispheres as a percentage of ipsilateral damage, using the formula ([C-I]/C)*100, where C is the mean of the contralateral area and I is the mean value of the ipsilateral area for each brain sample.

Brain damage in the different groups was microscopically evaluated by analyzing the CA 1, CA 2–3 and dentate gyrus (DG) of the hippocampus and the parietal cortex (CTX), with an Olympus BX 50 light microscope (x400). Brain injury was estimated from several cresyl violet histological sections of brains using a semi-quantitative histopathological scoring system, which was a modification of that reported by Hedtjärn et al. (2002) [43]. Evaluations were carried out by a researcher who was blind to the identity of the group being evaluated. Injury in the parietal cortex was graded from 0 to 4 (0 = no observable injury; 1 = a few small isolated groups of injured cells; 2 = several larger groups of injured cells, mild infarction; 3 = moderate confluent infarction; 4 = extensive confluent infarction encompassing most of the hemisphere). Damage in the hippocampus was assessed in terms of both hypotrophy (shrinkage; score of 0–3) and observable cell injury/infarction (0–3) in each of the three regions studied, resulting in a histopathological score ranging from 0 to 6. Thus, the maximum score for the hippocampus could reach 18 points. Finally, the total score graded from 0 to 28, was the sum score for all four regions and macroscopic evaluations, taking into consideration if the lateral ventricle was symmetric (= 0) or asymmetric (= 1) and if the ipsilateral one was dilated slightly (= 1), moderately (= 2) or severely (= 3).

#### Reactive astrogliosis

In order to evaluate astrogliosis, brains were removed and immersion-fixed in 4% paraformaldehyde and then stored in 30% sucrose until they sank. Coronal sections were cut at a thickness of 60 μm at at interaural distance 5.40 mm and bregma -3.60 mm level. Slices were washed three times in PBS and incubated for 15 minutes in blocking solution (0.25% Triton X-100 in 0.5% BSA in PBS). Sections were incubated overnight at 4°C with a monoclonal rabbit primary antibody to glial fibrillary acid protein (GFAP) (1:1000, Dako, Denmark) diluted in 0.25% Triton X-100 in 0.5% BSA in PBS. The slides were washed three times in PBS and incubated for 1 hour in an anti-rabbit secondary antibody conjugated with Alexa 488 (1:200; Invitrogen) and counterstained with DAPI (1:1000; Invitrogen). Negative controls received the same treatment omitting the primary antibodies and showed no specific staining. Fluorescently immunolabeled sections of whole brain were analyzed using an Olympus Fluoview FV500 Confocal Microscope, taking photos of the CA 1 and dentate gyrus areas of the hippocampus.

#### Assessment of white matter injury

Slices were dewaxed, rehydrated, washed two times in PBS and treated for 15 minutes with hydrogen peroxide (H_2_O_2_) (1%) in PBS to inactivate endogenous peroxidases, and then rinsed thoroughly in PBS to completely eliminate H_2_O_2_. Subsequently, the sections were incubated for 10 minutes in a blocking solution (0.25% Triton X-100 in PBS) and after washed twice in 0.5% BSA in PBS. Sections were incubated overnight at 4°C with a mouse monoclonal primary antibody to MBP (1:100, Santa Cruz Biotechnology, CA, USA) diluted in 0.25% Triton X-100 and 0.5% BSA in PBS. After immunostaining, the slides were washed three times in PBS and incubated for 1 hour with peroxidase-labeled second antibody at a dilution of 1:100 (HRP anti-mouse, Santa Cruz Biotechnology, CA, USA). Finally, the sections were stained with diaminobenzidine, counterstained with hematoxylin (Sigma-Aldrich Co. Ltd., Gillingham, UK), dehydrated in ethanol, cleared in xylene, and coverslipped with DPX mounting medium (Sigma-Aldrich Co. Ltd., Gillingham, UK). Immunolabeled sections of different areas of the brain were analyzed using an Olympus BX 50 light microscope. White matter integrity was analyzed by measuring the density of MBP immunostaining, using a computerized video-camera-based image-analysis system (Image J software) according to Liu et al. (2002). Evaluations were performed by a researcher who was blind to the identity of the group being evaluated. Unaltered TIFF images were digitized, segmented (using a consistent arbitrary threshold of -50%), and binarized (black versus white). The total number of black pixels per hemisphere was counted, and average values were calculated per brain, and expressed as pixels per hemisphere. Hemisphere areas were also outlined and measured for each section that was analyzed by densitometry. At least three sections per brain were analyzed and only sections with technical artifacts related to the staining procedure were excluded. Densitometric values were expressed as ratios of ipsilateral-to- contralateral hemispheric measurements (I:C).

### Assessment of mitochondrial state

#### Tissue collection

For flow cytometry studies, three experimental groups (control, HI and RVT-b) (n≥5) and three different points of time after hypoxic-ischemic insult (0 h, 3 h and 12 h) were evaluated. After flushing with Ringer lactate solution, the non-fixed tissue samples from fresh ipsilateral brain regions were disaggregated in collagenase (Invitrogen, The Netherlands) solution (1,5 mg/ mL) in Hanks' Balanced Salt solution (HBSS; Sigma-Aldrich, St Louis, Mo, EEUU) at 37°C for 20 min and further separated using a cell strainer. Then, cell suspensions were incubated with different fluorochromes and conjugates. Different analyses were performed using an EPICS ELITE Flow Cytometer (Colter, Inc., Florida, USA). To exclude debris and cellular aggregates, samples were gated based on light scattering properties, in side scattering (SSC), which correlates with cell complexity, and forward scattering (FSC), which correlates with cell size, and 10,000 events per sample within a gate (R1) were collected. Events within R1, which corresponded to individual cells, were analyzedfor their fluorescence. An unstained sample was used as a control to determine levels of autofluorescence. Data analysis was performed using the Summit v4.3 software.

Prior to the study by flow cytometry studies, we analyzed the viability in cell suspensions (live cells vs debris) and evaluated cellularity (cell aggregates and individual cells), excluding debris and cell aggregates. Then, when analyzing samples by flow cytometry, we first analyzed unstained samples from each animal, serving as a negative control to correct for autofluorescence. In this sense, we obtained similar values of autofluorescence for all samples, which were used to establish the same fluorescence gating for all samples in order to exclude autofluorescence.

#### Mitochondrial inner membrane integrity

The level of cardiolipin was determined by using the fluorochrome Nonyl Acridine Orange (NAO, Invitrogen, The Netherlands). This marker binds to cardiolipin that is located in the mitochondrial internal membrane and is essential for protein functionality and ATP synthesis. 750 μl of cell suspensions (1x10^6^ cells/mL) were incubated with 4 μl NAO (10^−2^ M) in PBS at 4°C and in the dark conditions for 30 minutes and later cells were washed twice in buffer. Samples were immediately evaluated using flow cytometry. Only integral cells were quantified by means of the NAO method, and therefore detritus and/or fragmented cells were not measured.

#### Mitochondrial transmembrane potential

Mitochondrial transmembrane potential was analyzed using Rhodamine 123 (Rh123, Invitrogen, The Netherlands), a lipophilic cationic fluorochrome, which accumulates inside the mitochondria in proportionto the mitochondrial transmembrane potential. A decrease in the fluorescence of Rh 123 indicates a loss of mitochondrial transmembrane potential. The cell suspension was incubated with Rh123 (4 μl/100 ml) in HBSS for 30 min at 37°C, followed by washing and incubation in HBSS for 30 min; cells were subsequently washed twice in buffer. Finally samples were analyzed using the flow cytometer.

#### Reactive oxygen species

Intracellular ROS formation was detected using fluorochrome 2′,7′-dichlorofluorescein diacetate (DCFH-DA, Invitrogen, The Netherlands). This probe is cell-permeable and is hydrolyzed intracellularly to the DCFH carboxylate anion that is retained in the cell. Two-electron oxidation of DCFH results in the formation of a fluorescent product, dichlorofluorescein (DCF), which can be monitored by flow cytometry. Cell suspensions were incubated with DCFH-DA fluorochrome (4 μl/100 ml) in HBSS for 30 min at 37°C and were later washed twice in buffer before loading into the flow cytometer.

### Behavioral impairment evaluation

On P90, we evaluated hypoxia-ischemia induced behavioral impairments by using the open field, hole-board, T-maze and novel object recognition tests in control, HI and RVT-b groups. All tests were performed by a researcher who was blind to the group to which the animals pertained.

#### Open field test

The open-field consists of a rectangular container made of dark polyethylene (60×60×30 cm) to provide optimal contrast to the white rats in a dimly lit room. The base of the cage is divided by lines in peripheral squares (12) and central squares (4). Testing was conducted in a silent room with constant light (300 lux). Rats were individually placed in the center of the apparatus to initiate a 30 minutes test session. Each session was recorded with a video camera and directly analyzed with the SMART (Spontaneous Motor Activity Recording & Tracking) v.3.0 software system (Panlab, Barcelona, Spain). Total, central and peripheral covered distance (mm), velocity (mm/s) and the time spent in both areas were analyzed.

#### Hole-board test

The hole-board test was carried out using a grey iron plate covered with dark formica (62×62×36 cm) with a raised floor insert (7 cm above the floor) with 16 holes, each with a diameter of 5 cm. Each rat was placed in the center of the apparatus and left to explore the arena for five minutes (Lee et al., 2014). The frequency of and time spent head dipping into the holes was recorded as a measure of neophobia. A head dip was scored when the head was introduced into the holes at least to the level of the eyes.

#### T-maze test

Working memory was tested using a T-maze alternation task. The experiments were performed in a T-maze constructed of wood and painted brown. The walls were 23 cm high, and the alleys were 18 cm wide. The length of the main alley was 31 cm, and the length of the side alleys was 31 cm. The side alleys were closed off from the main alley by movable doors. A week before habituation, all animals were partially food restricted (each female received 15 g of food per day and each male received 20 g of food per day) and remained that way throughout the remaining part of the experiment. This maintained each animal above 85% of its free-feeding body weight. The T-maze was cleaned between different animals but not between different trials. The food reward was a 5 g food pellet. The full experiment consisted of three parts: habituation, training, and testing. During habituation, all animals were placed on the T-maze until they ate two pieces of food or 90 s had elapsed. This was repeated three times a day for five days. During training, all animals underwent six trials a day. Each trial consisted of two runs: a forced run and a free run. On the forced run, rats were forced to obtain a piece of food from one goal arm of the T-maze, with the other goal arm blocked by its door. Animals were then placed back into the start arm for a 10 s delay period. At the beginning of the free run, the rats were allowed to choose either goal arm. If the rats chose the arm opposite the one they had been forced into during the forced run, they received the food reward. If the rats chose the same arm into which they had been forced, they received no food reward. There was a 5 minutes inter-trial interval. The training period ended after control animals made 70% correct choices on two consecutive days. Rats were then tested for their performance at 10 or 40 s delay periods. Rats were given three 10 s delay and three 40 s delay trials during the day of testing. The sequence of delays and forced-run food locations (left or right) were randomized each day, with the stipulation that the same delay or the same forced-arm location could not be used for three trials in a row. Rats were then tested for their performance in the maze recording the number of correct entries. Goal entries were defined as the placing of the four paws in the arm.

#### Novel object recognition test

This test is a nonrewarded paradigm that measures visual episodic memory. Animals were first habituated to the experimental room for a 30 min period. On the first day, animals were habituated to the apparatus (15x28x50 cm) for a 10 min period. On the next two days, animals were allowed to freely explore two identical novel objects for a 10 min period. On the test day, one of the objects was replaced by a new different one and the animal was allowed to freely explore for 10 minutes. Exploratory behavior was scored for investigation time of each object in the test session. The discrimination index ([time in new object minus time in familiar object] / [time in new object plus time in familiar object]) was defined as the parameter for evaluation.

### Statistical analyses

All data were expressed as the mean ± standard error of the mean (SEM), and were analyzed using a one-way analysis of variance followed by Bonferroni-Dunn correction. Statistical analysis was performed using GraphPad Prism version 5 (Graph Pad Software, San Diego, CA, USA).

## Results

### Resveratrol pretreatment but not post-treatment protected against brain injury

**Fig 1:**
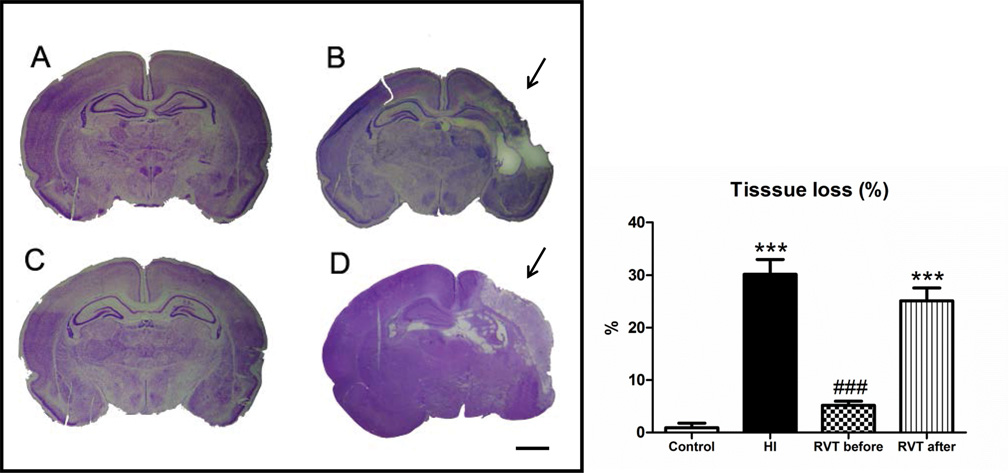
Brain tissue loss induced by hypoxia-ischemia in 7 day-old rats and evaluated at P14. Representative stereomicroscopic photographs of 5 μm Nissl-stained brain sections (interaural distance 5.40 mm and bregma -3.60 mm) are shown. (**A**) Control group with normal morphological brain (n = 8). (**B**) HI brain with evident loss of tissue in the ipsilateral side of the cortex, and with obvious damage to the hippocampus (region indicated by arrows) (n = 10). (**C**) Brain treated with resveratrol 10 minutes before hypoxia, which is similar to the control brain (n = 10). (**D**) HI brain treated with resveratrol immediately after hypoxia, with obvious signs of infarct, as denoted by the arrow (n = 10). Scale bar: 2.5 mm. The histogram illustrates percentage of tissue loss is expressed. Asterisks denote the significance levels when compared to the control group (*** *P*<0.0001). The hash denotes the significance levels when compared to the HI group (^###^ *P*<0.0001).

Representative photographs of coronal sections of the perinatal brains of the different experimental groups are shown in Fig 1. Retraction of the ipsilateral hippocampus and loss of cortical volume as a consequence of the increased ventricular size were characteristics of the HI group in P14 animals. In general these brains revealed an infarct area on the ipsilateral side with loss of brain tissue (Fig 1B), while no macroscopic differences could be observed between control (Fig 1A) and animals treated with resveratrol 10 minutes before hypoxia (Fig 1C). However, animals receiving resveratrol immediately after hypoxia showed an infarct area which was similar to that of the hypoxia-ischemia group (Fig 1D).

A quantitative evaluation of infarct area is shown in Fig 1. The HI group was found to have a high percentage of damage (30.1±2.4%) in comparison to control (0.9±1.2%), while no statistical difference was found between the RVT-b (5.1±0.6%) and control groups ([F(3,34) = 62.24, *P* < 0.0001]). No signs of infarct could be observed in resveratrol pretreated animals, but in the RVT-a group, a significant increase in the affected area of the brain (25.1±2.2%) was found, with values similar to those of the HI group. The mortality rate was 10% in the HI group, 8% in the RVT-a group, and 0% in control and RVT-b groups.

**Fig 2:**
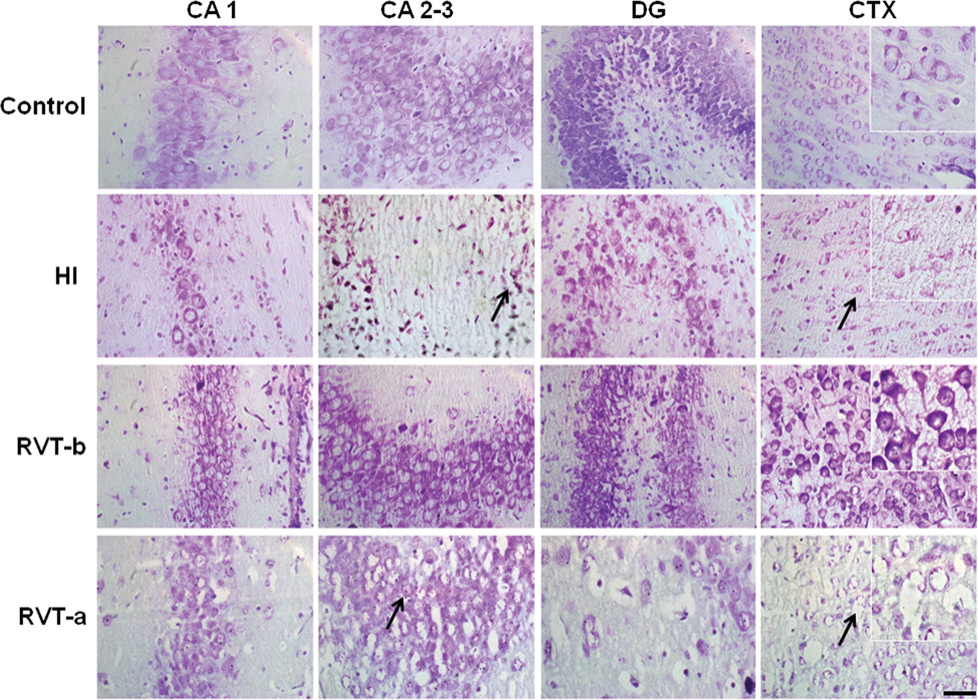
Representative microphotographs of Nissl-stained brain sections in animals exposed to hypoxia-ischemia at P7 and evaluated at P14. Individual fields represent different experimental groups and different areas of the hippocampus (CA 1, CA 2–3 and DG) and from the parietal cortex (CTX), with high magnification insets in CTX. Cell loss was especially evident in the DG and parietal cortex (arrows) in the hypoxia-ischemia and resveratrol post-treatment groups. In contrast, the resveratrol pre-treated group showed a remarkable conservation in the cellularity of the different studied areas with respect to the HI group. Scale bar: 50 μm.

The hippocampus and the parietal cortex of hypoxic-ischemic animals displayed significant evidence of infarction, whereas those of control animals did not. In Nissl stained brain sections, hypoxia-ischemia induced a significant cell loss (see arrows in Fig 2). Moreover, asphyctic animals showed swollen and deformed neurons especially in the ipsilateral CA 1 and CTX areas. In contrast, only mild cell loss and a few damaged neurons were observed in slices in all brain regions studied from animals pretreated with resveratrol, Overall, the subfields in the hippocampus and the parietal cortex were similar in structure to those of the control group. Animals treated with resveratrol immediately after hypoxia presented extensive cell loss, as well as swollen and deformed cells, with overall characteristics which were similar to those exhibited by the hypoxia-ischemia group.

**Fig 3:**
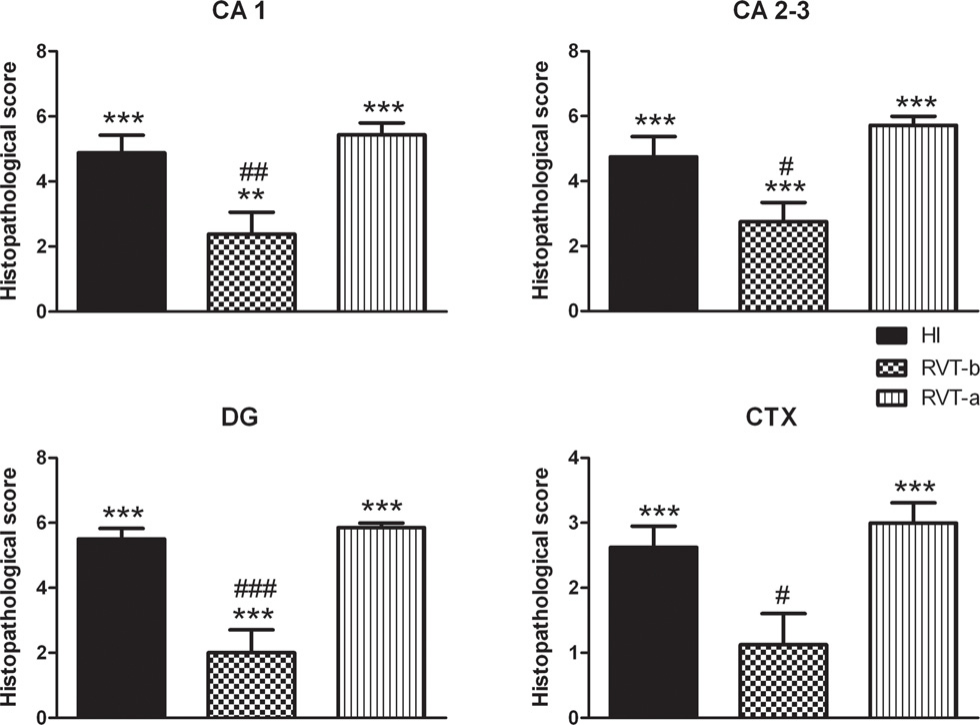
Histopathological score of damage in P14 rat brains of different groups expressed as the mean ± SEM. Asterisks denote the significance levels when compared to the control group (*** *P*<0.0001). The control group (n = 11) is not shown, because it histopathological score for all areas was 0. The hash symbols denote the significance levels when compared to the HI group (^###^ *P*<0.0001). It can be clearly seen that the group pretreated with resveratrol (n = 8) had a lower histopathological score compared with the HI group (n = 8). This was clearly not the case for the post-resveratrol treated group (n = 8).

Results from histopathological scoring (Fig 3) corroborate those seen under microscopy (Fig 2). Thus, animals that received resveratrol before hypoxia demonstrated significantly lower values of neuropathology in the CA 1, CA 2–3, DG and CTX, than those of the HI group; CA 1 ([F(3,30) = 35,18, *P* < 0.0001]), CA 2–3 ([F(3,30) = 39.49, *P* < 0.0001]), DG ([F(3,30) = 61.43, *P* < 0.0001]) and CTX ([F(3,30) = 22.75, *P* < 0.0001]). This improvement was more evident in the dentate gyrus and CA 1, with values being similar to those of controls (control group not shown, because its histopathological score for all the areas was 0). In particular, the RVT-b group got significantly lower score in the whole hippocampus (the sum of each hippocampal area) (7.125) in comparison to the same summed area of the HI group (15.13) and RVT-a group (17). The overall, total score was also significantly (*p*<0.0001) reduced in the RVT-b group, with 9.25 points versus the 22.25 and 25 points of the HI and RVT-a groups, respectively. In contrast, when resveratrol was administered immediately after the injury, no neuroprotective effects in terms of an improved histopathological score. This assessment of the brain injury indicates that resveratrol pretreatment but not post-treatment reduced infarct volume and attenuated cell damage.

### Pretreatment with resveratrol minimized the astroglial reactive response

**Fig 4:**
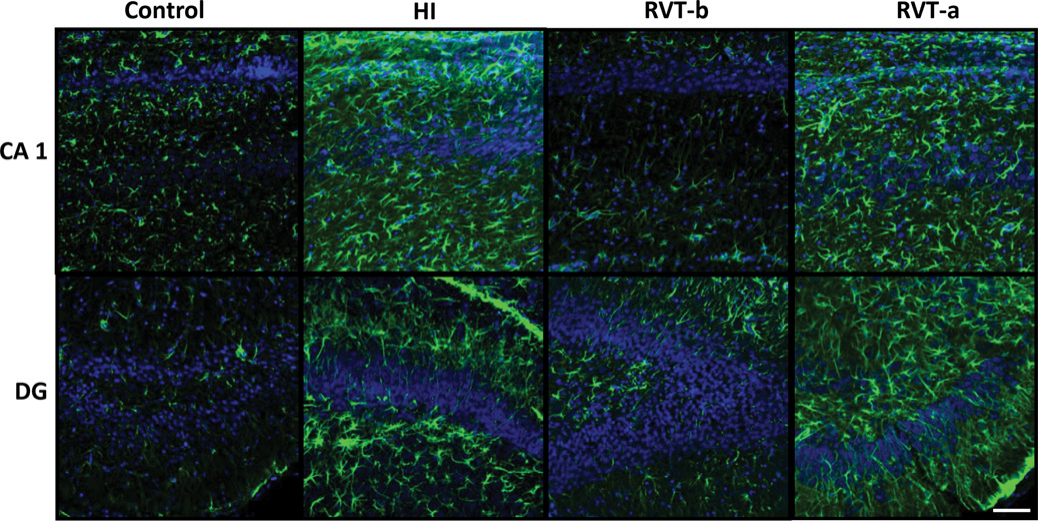
Representative confocal microphotographs of glial fibrillary acidic protein (GFAP)-immunoreactivity in brain sections counterstained with DAPI. On postnatal day 14, GFAP immunoreactivity (green) was particularly pronounced in the vicinity of damaged areas and this reactivity was substantially reduced in animals pre-treated with resveratrol. Scale bar: 40 μm.

GFAP astrogliosis was found in animals with hypoxic-ischemic injury, particularly in regions near dead or dying cells, such as the CA1 and dentate gyrus areas of the hippocampus, while control cases showed low levels of GFAP immunoreactivity (Fig 4). This reactive astrocyte response was diminished when resveratrol was administered before hypoxia, but not after it, demonstrating that pretreatment with resveratrol reduced the astroglial reactive response.

### Resveratrol pretreatment preserved myelination

**Fig 5:**
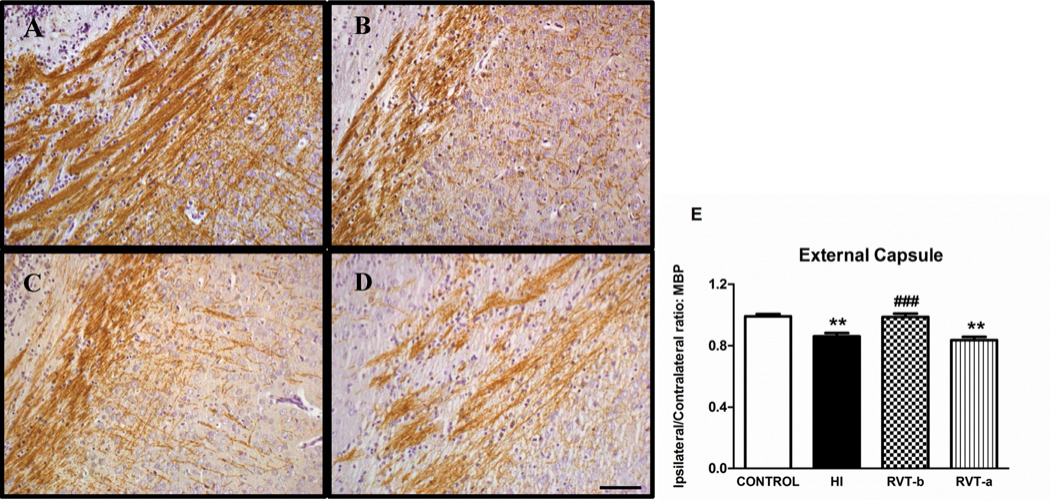
Representative light microphotographs of myelin basic protein (MBP)-stained brain sections and comparison of loss of MBP immunostaining in the external capsule of different groups: 14-day-old control (n = 5) (A), HI (n = 14) (B), RVT-b (n = 14) (C) and RVT-a (n = 7) (D) (scale bar: 40 μm). In the histogram (**E**), the extent of tissue injury, expressed as a ratio of left-to-right hemispheric MBP immunostaining is represented. Asterisks denote the significance levels when compared to the control group (** *P*<0.0001). Hashes denote the significance level when compared to the HI group (^###^ *P*<0.0001).

**Fig 6:**
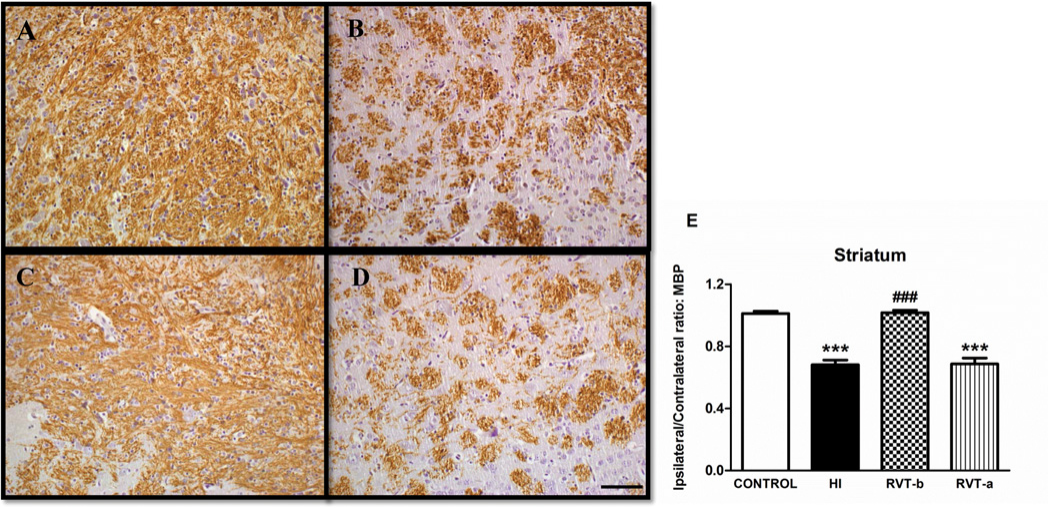
Representative light microphotographs of myelin basic protein (MBP)-stained brain sections and comparison of loss of MBP immunostaining in the striatum of different groups: 14-day-old control (n = 6) (A), HI (n = 15) (B), RVT-b (n = 13) (C) and RVT-a (n = 7) (D) (scale bar: 40 μm). The histogram (**E**) illustrates the extent of tissue injury associated with the various treatments and values are expressed as a ratio of left-to-right hemispheric MBP immunoreactivity levels. Asterisks denote the significance levels when compared to the control group (***P<0.0001). Hashes denote the significance levels when compared to the HI group (^###^ *P*<0.0001).

A substantial loss of ipsilateral MBP immunostaining was observed in both external capsule and striatum in P14 animals that had been subjected one week earlier to hypoxia-ischemia with respect to controls (Figs 5 and 6). This loss was absent with resveratrol pretreatment, but not when resveratrol was administered after the injury.

Quantitative analysis corroborated these initial impressions from microscopy observation. At the level of the external capsule (Fig 5), a substantial loss of ipsilateral MBP immunostaining ([F(3,36) = 11.63, *P* < 0.0001]) was observed in the HI group (0.86) when compared with control animals (0.99). Pups pretreated with resveratrol showed a smaller degree of MBP loss in the ipsilateral hemisphere (0.98), whereas this improvement was not apparent in the RVT-a group (0.83) whose MBP (I:C) ratio was similar to that observed in the HI group.

At the level of the mid-striatum (Fig 6), HI neonatal rats (0.68) exhibited a significant ([F(3,37) = 48.49, *P* < 0.0001]) loss in MBP immunostaining in subcortical white matter when compared with control animals (1.01). Resveratrol-pretreated animals showed a lesser degree of MBP loss in the ipsilateral hemisphere (1.01), with a similar MBP ratio (I:C) to that observed in the control group. Additionally, there was also a significant reduction in the I:C immunostaining ratio in animals who received resveratrol after injury (0.68). These results suggest that resveratrol when administered as a preventive agent is able to ameliorate the loss of myelination.

### Assessment of mitochondrial state

#### Mitochondrial inner membrane integrity was protected by resveratrol

**Fig 7:**
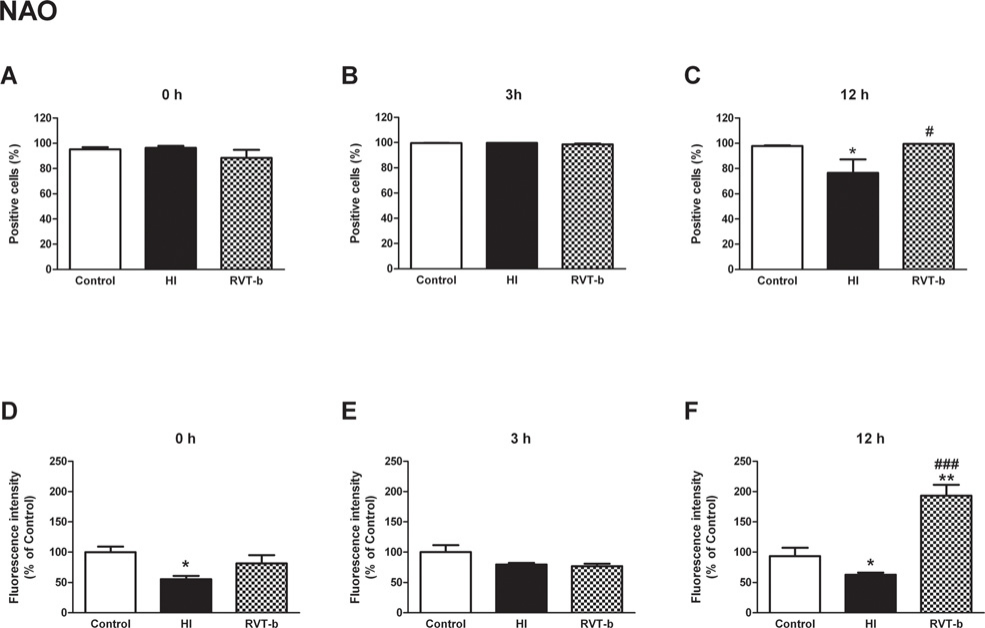
Mitochondrial inner membrane integrity evaluation in suspension of acutely isolated cells using nonyl acridine orange (NAO). **Percentage of NAO-positive cells at different time points after hypoxia-ischemia**: (**A) 0 h, (B) 3 h and (C) 12 h. Relative fluorescence intensity of cells with *in vivo* marker NAO at different time points after hypoxia-ischemia**: **(D) 0 h, (E) 3 h and (F) 12 h**, in control (n≥5), HI (n≥5) and animals pretreated with resveratrol (n≥5). Asterisk denotes the significance levels when compared to the control group (* *P*<0.05). The hash symbol denotes the significance levels when compared to the HI group (^#^ *P*<0.05).

Integrity of the inner mitochondrial membrane was evaluated using the fluorochrome NAO. The initial states, 0 h and 3 h, showed no statistically significant differences among all groups (Fig 7A and 7B). However, at 12 h ([F(2,20) = 6.006, *P* < 0.05]), the percentage of NAO positive cells significantly decreased in the HI group (76.53±10.71%) with respect to the control group (97.37±0.61%), suggesting reduced mitochondrial integrity, while animals pretreated with resveratrol maintained mitochondrial inner membrane integrity (99.42±0.06%), and showed similar values in comparison to the control group (Fig 7C).

Nevertheless, we found that at initial time, 0 h ([F(2,21) = 5.024, *P* < 0.05]), the HI group underwent a diminishment with statistical differences in the relative values of fluorescence intensity for NAO (55.52±5.39%) with respect to the control group, but the RVT-b group did not show a significant reduction(81.65±13.55%) (Fig 7D). At 3 h there was no significant difference among the groups (Fig 7E). In contrast, at 12 h ([F(2,15) = 8.06, *P* < 0.05]) after resveratrol administration the relative values of NAO fluorescence intensity were significantly higher (193.2±17.93%) than in the HI group (62.6±3.69%) and even in the control group (Fig 7F). These data indicate that resveratrol protected the integrity of the mitochondrial inner membrane.

#### Evaluation of mitochondrial transmembrane potential

**Fig 8:**
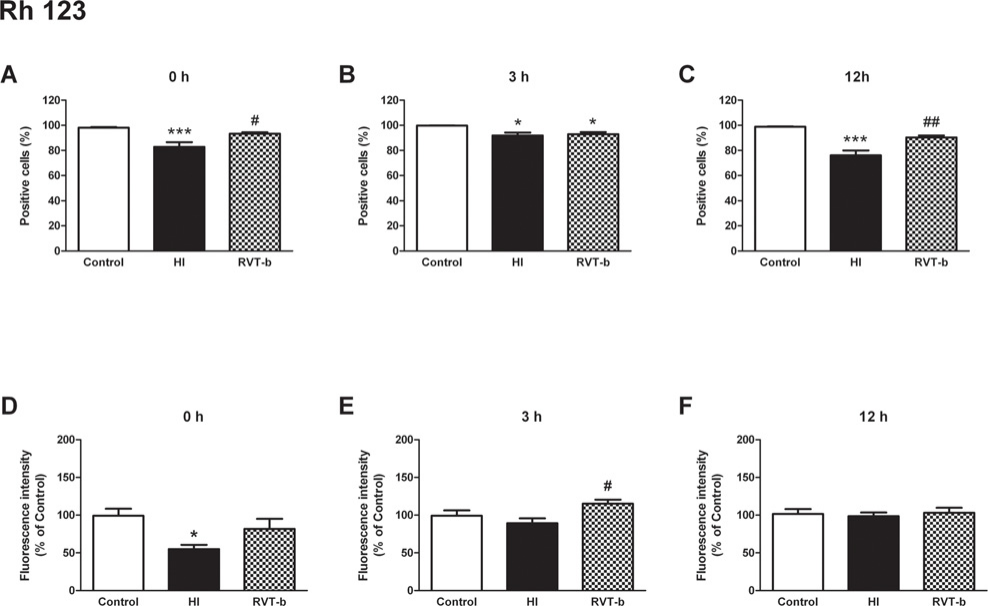
Analysis of mitochondrial transmembrane potential by Rhodamine 123 in suspension of acutely isolated cells. **Percentage of cells labeled with the *in vivo* marker Rh 123 at different time points after hypoxia-ischemia**: (**A**) **0 h, (B) 3 h and (C) 12 h. Relative fluorescence intensity of cells exhibiting Rh 123 fluorescence at different time points after hypoxia-ischemia**: (**D) 0 h, (E) 3 h and (F) 12 h,** in control (n≥5), HI (n≥5) and animals pretreated with resveratrol (n≥5) groups. Asterisks denote the significance levels when compared to the control group (*** *P*<0.0001 or * *P*<0.05). The hash symbols denote the significance levels when compared to the HI group (^##^ *P*<0.005 or ^#^ *P*<0.05).

Mitochondrial transmembrane potential was analyzed using the fluorochrome Rhodamine 123. At 0 h ([F(2,19) = 10.8, *P* < 0.005]), hypoxia-ischemia generated a decrease in the percentage of Rh 123 positive cells (82.89±3.78%), in comparison to the control group (98.14±0.47%). In contrast, the RVT-b group showed a percentage of Rh 123 positive cells (93.25±1.18%) which was similar to that of the control group (Fig 8A). At 3 h ([F(2,12) = 6.84, *P* < 0.05]), the number of Rh 123 positive cells diminished in HI animals (91.99±2.28), and also in the resveratrol pretreated group (92.91±1.71%). Although these reductions were statistically significant, they were not as evident as the differences at 0 h with respect to control (99.88±0.03%) (Fig 8B). However, at 12 h ([F(2,24) = 28.02, *P* < 0.0001]) animals subjected to the hypoxic-ischemic event underwent again an important diminishment in the percentage of Rh 123 positive cells (76.08±3.91%) in comparison to the control group (98.81±0.37%), while animals pretreated with resveratrol showed similar values (90.39±1.59%) to those of the controls (Fig 8C).

**Fig 9:**
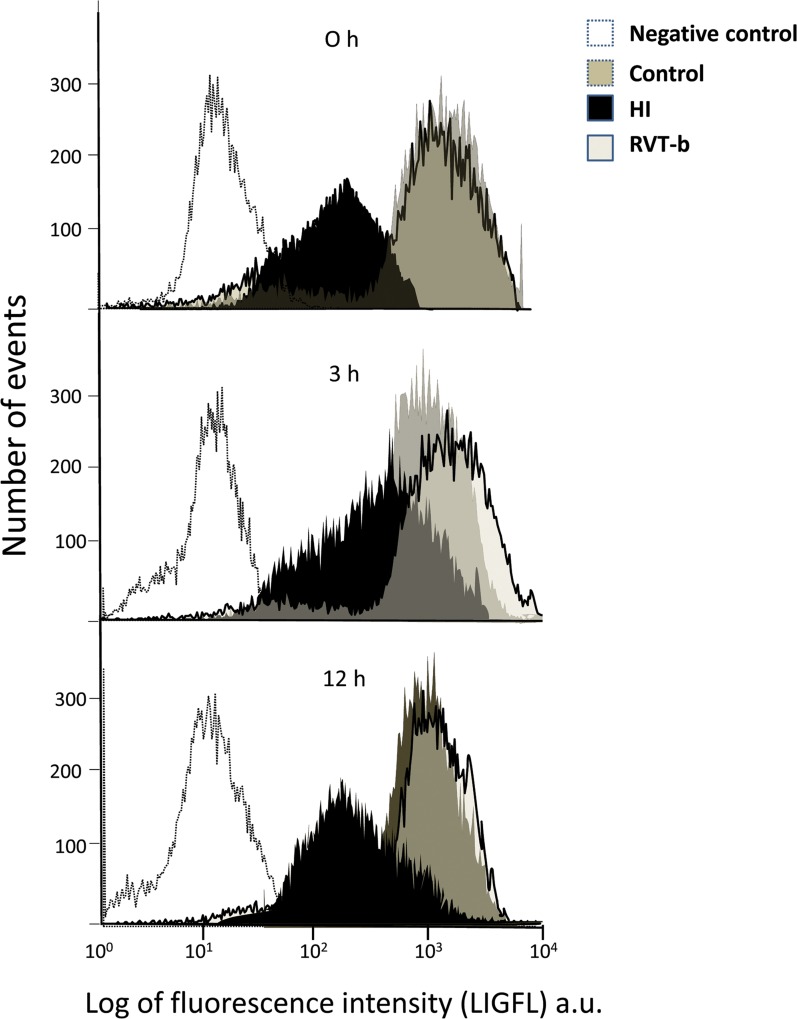
Representative fluorograms obtained after flow cytometry analysis showing mitochondrial transmembrane potential measured as Rh 123 fluorescence at different points of time after hypoxia-ischemia (0 h, 3 h and 12 h) in control, HI and animals pretreated with resveratrol. The x-axis represents the number of events and the y-axis represents the values of fluorescence intensity in logarithm values. The negative control consisted of unstained samples from each animal to remove the autofluorescence.

At 0 h ([F(2,21) = 5.088, *P* < 0.05]), the relative values of Rh123 fluorescence intensity in cells isolated from HI rats were reduced (55.13±5.44%), whereas in those from animals pretreated with resveratrol, the observed values (81.75±13.34%) were found to be similar to those of the control group (Fig 8D). In contrast, at 3 h ([F(2,12) = 4.174, *P* < 0.05]) there was not a significant difference between control and HI groups, but animals that received resveratrol exhibited higher values (115.2±13.34%) than the HI group (89.2±6.52%) (Fig 8E). At 12 h there was no significant difference among the groups (Fig 8F). Representative fluorograms of mitochondrial transmembrane potential measured ash Rh 123 fluorescence also revealed that HI animals had lower values of fluorescence than control and treated animals (Fig 9). These results provide evidencethat resveratrol maintainsd mitochondrial transmembrane potential.

#### Resveratrol reduced the production of oxygen reactive species

**Fig 10:**
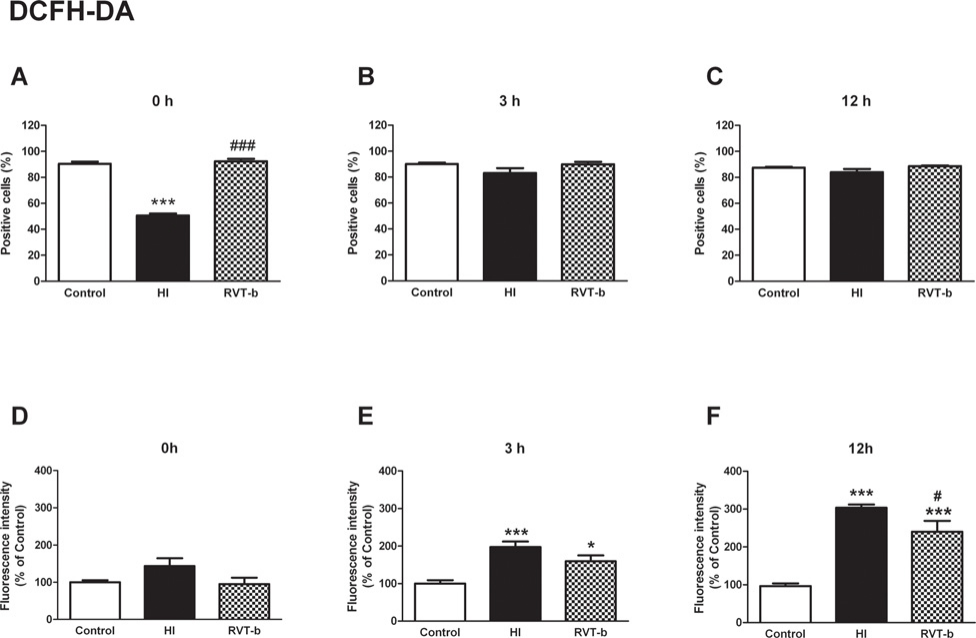
Effect of brain hypoxia-ischemia on the production of reactive oxygen species in suspension of acutely isolated cells, measured using DCFH-DA. **Percentage of DCFH-DA positive cells at different time points after hypoxia-ischemia**: (**A) 0 h, (B) 3 h and (C) 12 h. Relative fluorescence intensity of cells with *in vivo* marker DCFH-DA at different time points after hypoxia-ischemia**; (**D) 0 h, (E) 3 h and (F) 12 h**, in control (n≥5), HI (n≥5) and animals pretreated with resveratrol (n≥5) groups. Asterisks denote the significance levels when compared to the control group (*** *P*<0.0001 * *P*<0.05). The hash symbols denote the significance levels when compared to the hypoxia-ischemia group (^###^ *P*<0.0001 or ^#^ *P*<0.05).

Oxygen reactive species were detected using the DCFH-DA fluorochrome. At 0 h ([F(2,18) = 216,9, P < 0.0001]) while the HI group showed a decrease in the percentage of DCFH positive cells, the treated group presented similar percentage values which were statistically similar to those of the control group (Fig 10A). At 3 h and 12 h, no statistically significant differences were found among all the groups (Fig 10B and 10C).

Concerning the relative values of fluorescence intensity indicative of ROS production, at 0 h no significant difference were found among all the groups (Fig 10D). At 3 h ([F(2,13) = 12.84, *P* < 0.0001]) there was an increase in HI rats (197.19±14.93%) and also in RVT-b animals (159.35±15.64%), although this was not statistically significant (Fig 10E). At 12 h ([F(2,18) = 67.58, *P* < 0.0001]) the relative values of fluorescence intensity of DCFH were significantly increased in the HI (303.7±8.58%) and RVT-b (240.422±28.73%) groups with respect to the control group, but this increase was less pronounced in the RVT-b group (Fig 10F). These results provide evidence that resveratrol reduced ROS production in living cells.

### Evaluation of behavioral impairment

Behavioral deficits were evaluated on day P90 in HI and control animals. Afterwards, we tested if administration of resveratrol 10 min before hypoxia could improve behavioral impairments.

#### Hypoxic-ischemic injury did not alter motor activity

**Fig 11:**
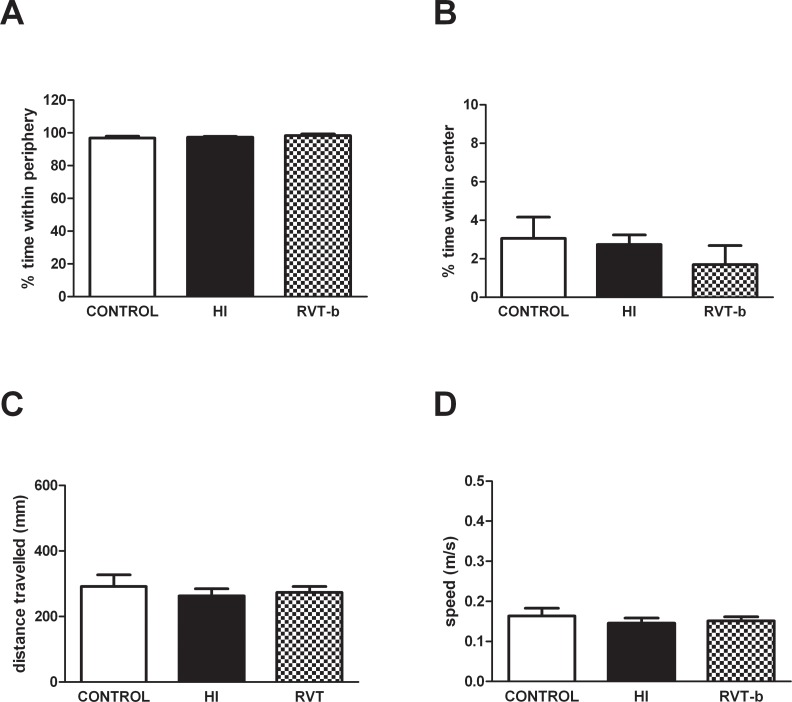
Evaluation of spontaneous locomotor activity in the open field test performed at P90 in control (n = 16), HI (n = 14) and RVT (20 mg/kg) treated animals (n = 10). Evaluated parameters were (**A**) % time in the periphery, (**B**) % time in the center, (**C**) total distance travelled and (**D**) speed in the open field.

No significant differences were found between control, HI and RVT-b (20 mg/kg) groups in the percentage of time spent in the periphery (Fig 11A) or in the center of the apparatus (Fig 11B). In the same way, no changes were observed in the total distance travelled (mm) (Fig 11C) or speed (m/s) (Fig 11D), demonstrating that hypoxic-ischemic injury did not alter motor activity.

#### Resveratrol reduced anxiety and neophobia induced by hypoxia-ischemia

**Fig 12:**
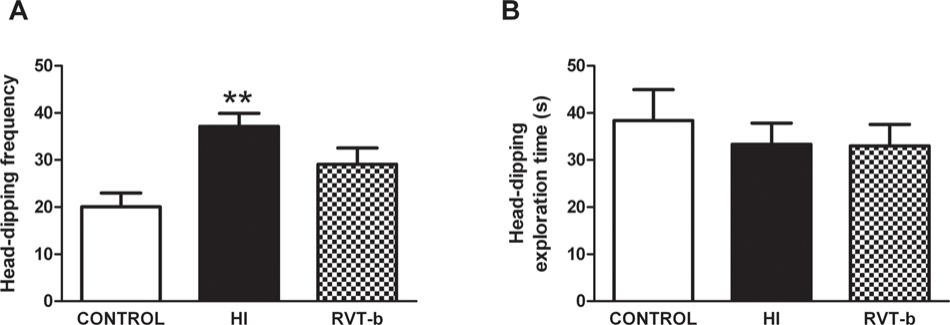
Effect of neonatal hypoxia-ischemia and resveratrol pretreatment on the hole-board test performed at P90. (**A**) The frequency and (**B**) the time spent head-dipping into the holes were recorded in control (n = 16), HI (n = 14) and RVT (20 mg/kg) treated animals (n = 10). Asterisks denote the significance levels when compared to the control group (**P < 0.005).

Rats that underwent hypoxia-ischemia showed a statistically significant increase in the frequency of head-dipping behavior ([F(2,37) = 9.114, *P* < 0.005], when compared to control animals (Fig 12A). In contrast, RVT (20 mg/kg) pretreated animals presented similar values to non-ischemic, control rats, suggesting that RVT is able to reduce the anxiety and neophobia associated with hypoxic-ischemic injury. No differences were found between groups in the hole-board test (HBT) for exploratory time (Fig 12B).

#### Resveratrol pretreatment improved spatial working memory impairments

**Fig 13:**
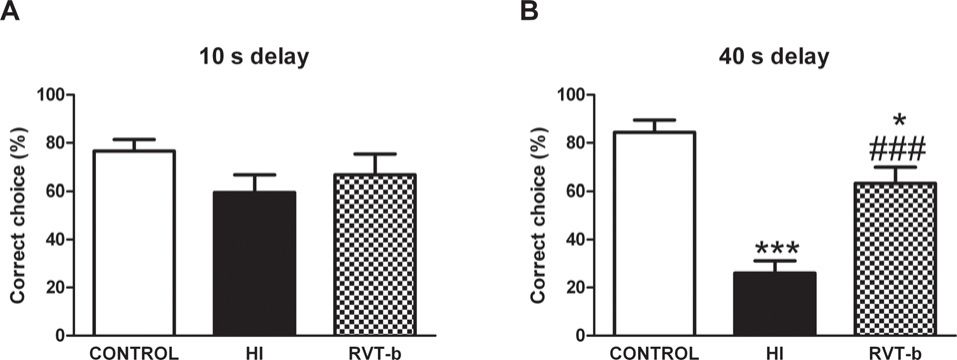
Effect of neonatal hypoxia-ischemia and pretreatment with resveratrol on choice accuracy in the discrete-trial delayed spatial alternation task (T-maze) in adult animals on P90. (**A**) Control (n = 16) and HI rats (n = 14), as well as RVT pretreated animals (n = 10) made a similar number of correct choices in the T-maze at 10 s delay. (**B**) In contrast, HI animals made significantly fewer correct choices after the 40 s delay. Impaired memory performance (percentage of correct trials) due to hypoxia-ischemia was reverted by RVT pre-administration. Asterisks denote the significance levels when compared to the control group (*** *P*<0.0001). The hash symbols denote the significance levels when compared to the HI group (^###^ *P*<0.0001).

At a 10 s delay interval (Fig 13A), no differences were found between groups. In contrast, at a 40 s delay interval ([F(2,37) = 32.57, P < 0.0001]), HI animals made significantly fewer correct choices (Fig 13B) when compared to control animals. RVT reversed these changes and significantly increased the number of correct choices when compared to HI rats. The results indicate a working memory dysfunction induced by hypoxia-ischemia which is prevented by the acute administration of RVT before the ischemic event.

#### Non-spatial working memory impairment was prevented by resveratrol

**Fig 14:**
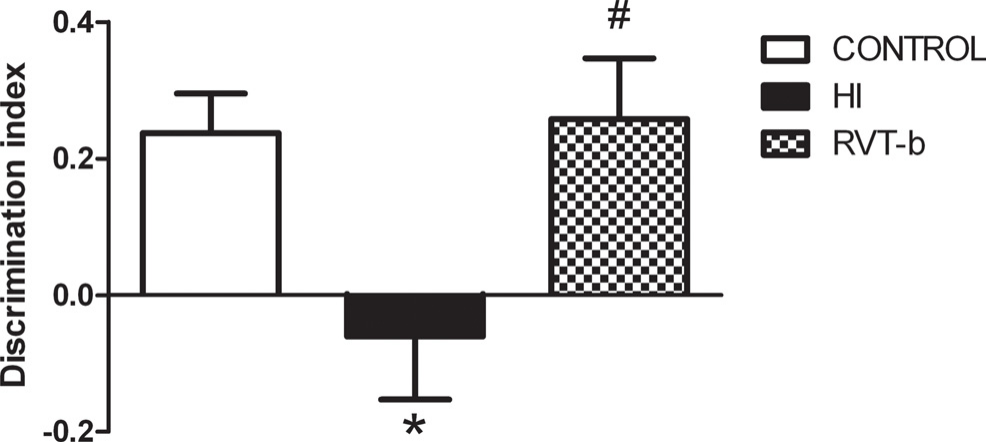
Effect of neonatal hypoxia-ischemia and treatment with resveratrol on the results of a novel object recognition test. On P90, HI adult animals (n = 14) displayed a decrease in discrimination index when compared to control animals (n = 16) that was fully reversed by resveratrol pretreatment (n = 10). Asterisk denotes the significance levels when compared to the control group (* *P*<0.05). The hash symbol denotes the significance levels when compared to the HI group (^#^ *P*<0.05).

Hypoxia-ischemia induced a significantly profound decrease in the discrimination index when compared to control animals (Fig 14) ([F(2,38) = 5.106, *P* < 0.05]). Acute resveratrol fully reverted the effects of hypoxia-ischemia on novel object recognition, recovering discrimination index to control values. Thus, resveratrol appears to prevent non-spatial working memory deficits induced by hypoxia-ischemia.

## Discussion

The aim of the present work was to characterize the effects of resveratrol administered either as a preventive agent or as a therapeutic agent in hypoxic-ischemic brain injury in neonatal rats. We have found that pretreatment with resveratrol, but not post-treatment, protected the brain from subsequent hypoxic-ischemic damage in different ways and that the neuroprotective effect of this polyphenol was long lasting, since behavioral outcomes were significantly improved in pre-treated ischemic animals when assessed at adulthood.

Resveratrol, when administered before the hypoxic-ischemic event, was found to be a potent protective agent that diminished tissue loss and consequently the infarct area, as reported by West et al. (2007) [41] and Karalis et al. (2012) [44]. Concerning astrocytes, reduced glial reactivity in the resveratrol pre-treated group suggests that the magnitude of the impact of the ischemic stress had been reduced. In this sense, although partial astrogliosis can confer neuroprotection by scavenging ROS and assisting with reconstruction following brain injury [45], excessive astrogliosis can result in the impairment of neuronal signaling and the disruption of myelination [46,47]. White matter damage and late cognitive impairment are important deleterious effects known to occur after hypoxic-ischemic injury in preterm children [48]. A widespread and complex inflammatory response to ischemia occurs which finally contributes to white matter injury [49], principally via the jeopardizing of oligodendroglia, since these cells are particularly susceptible to glutamate-mediated injury. The loss of MBP was absent when resveratrol was given before the injury, thus corroborating the findings of Karalis et al. (2011) [44].

Using immunohistological techniques, we did not observe any signs of neuroprotection with resveratrol administered after hypoxia-ischemia, in keeping with the findings of West et al. (2007) [41]. Consequently, and in order to avoid unnecessary animal testing, we decided not to test the efficacy of post-administered resveratrol in flow cytometry and long-lasting behavioral studies. Paradoxically, a protective effect of resveratrol given after hypoxic-ischemic injury has been reported by Karalis et al. (2011) [44]. However, this discrepancy is likely due to their using a shorter period (one hour) and to their use of higher doses of trans-resveratrol (90 mg/kg), since the neuroprotective effects of this stylbene are dose-dependent in this animal model [41,50,51] Thus, it is possible that resveratrol post-treatments can protect against mild hypoxic-ischemic injury, but not against severe or moderate injury. Similarly, when we administered resveratrol as a therapeutic agent, the damage produced by the deleterious hypoxia-ischemia cascade had already started and the dose of resveratrol which we used was unable to revert the initiated damage

Different studies have demonstrated the neuroprotective actions of resveratrol, including its anti-oxidative, anti-apoptotic and anti-inflammatory effects, in a variety of ischemia models [52–54]. These beneficial effects are known to be mediated via modulation of multiple signaling pathways [55,56]. Mitochondria play a fundamental role in the cascade triggered after hypoxic-ischemic injury, as mitochondrial respiration is suppressed [57]. In the developing brain, lack of oxygen results in a depletion of cellular energy reserves, which trigger several pathophysiological responses, but there is a common convergence at the level of the mitochondria [58]. Our results indicate that in the specific cell population which we isolated, resveratrol maintains a constant number of cells with an intact mitochondrial inner membrane and that those cells presented a high quantity of cardiolipin 12 hours after the injury. In the same way, when resveratrol was administered as a pretreatment the percentage of cells with intact transmembrane potential and the fluorescence intensity was similar to controls, whereas HI animals showed reduced values. This indicates that the cells that are alive maintain their transmembrane potential; in fact, rhodamine 123 accumulates specifically in the mitochondria of living cells [59].

As we have mentioned, oxidative stress is considered to be a major contributor to hypoxic-ischemic brain injury, and our data show that although there were fewer cells producing ROS in the HI group, those which were producing these species were doing so more vigorously This might be partially due to the fact that mitochondria are both a source and a target of ROS [23,60]; after the initial mitochondrial, an increase in the quantity of ROS production was observed to occur overtime. In contrast, this did not happen in the case of resveratrol pretreatment which ameliorates the production, as a result of its antioxidant effect. In addition to reducing the quantity of ROS production, we speculate that one of the ways resveratrol might protect against hypoxic-ischemic damage may be by protecting mitochondrial integrity and maintaining the membrane potential. Previous findings have demonstrated that resveratrol exerts one of its neuroprotective effects in this way [23], and also by modulating mitochondria [56], inducing mitochondrial biogenesis against brain ischemic stroke [61] and preserving brain mitochondria functions after hypoxia-reoxygenation [50].

One of the most significant findings of this study is the evidence that resveratrol when administered before hypoxia-ischemia, was able to improve the long-lasting cognitive deficits induced by hypoxia-ischemia, probably via the protection and preservation of neocortical and subcortical brain areas (sensorimotor cortex, hippocampus and striatum), and in particular, of neuronal networks responsible for learning and memory. The beneficial effects of resveratrol on cognition have been reported in other pathologies in rats such as epilepsy [62] and a stress model [63]. More importantly, this polyphenol has been shown to increase memory performance in primates and to increase hippocampal functional connectivity in older adults [64], in addition to playing an important role in enhancing cerebrovascular and cognitive functions in humans [65].

Hypoxia-ischemia did not seem to exert any obvious effects on basic motor abilities, corroborating the findings reported by Damoradan et al. (2014) [66]. This may partly be due to the high degree of cerebral plasticity of rats. Compensatory reorganization of vital functions following injury is possible and the contralateral hemisphere can functionally take over certain tasks from the injured hemisphere [67,68]. Anxiety-like behaviors observed in rats that underwent hypoxia-ischemia [69,70] were prevented by resveratrol. Head-dipping behavior in the HBT is considered to be a good index for evaluating the anxiety of rodents [71]. Impairments in reference and working memory evaluated in the T-maze performance were demonstrated after neonatal hypoxia-ischemia, as previously reported [,^68^, ^72^–^77^]. However, resveratrol prevented these memory deficits, as well as non-spatial working memory disruptions that were assessed by the novel object recognition test. This is based on the innate preference of rats to examine novel objects rather than familiar ones [72,78] and is primarily used to evaluate the effects of drugs on memory [79], not involving primary reinforcements or stressful cues, such as food deprivation and/or electric shocks [80–82].

## Conclusions

Our results provide novel evidence for the protective ability of resveratrol when administered before injury in the hypoxic-ischemic neonatal brain by ameliorating the morphological damage, especially in the cortex and in the hippocampus, and preventing rats from experiencing long-lasting functional damage. We postulate that one of the mechanisms by means of which resveratrol protects against hypoxic-ischemic damage may be the protection of mitochondrial inner membrane integrity and the maintenance of transmembrane potential, as well as the reduction of ROS production. A more profound understanding of the molecular mechanisms of action of this polyphenol in the hypoxic-ischemic model will require further investigation.

## Supporting Information

S1 Data Individual data for Fig 1. (PZF)

S2 Data Individual data for Fig 3. (PZF)

S3 Data Individual data for Fig 3 with control group. (PZF)

S4 Data Individual data for Figs 5 and 6. (PZF)

S5 Data Individual data for Fig 7 (Percentage of Positive Cells for NAO). (PZF)

S6 Data Individual data for Fig 7 (Relative Fluorescence Intensity for NAO. (PZF)

S7 Data Individual data for Fig 8 (Percentage of Positive Cells for Rh 123). (PZF)

S8 Data Individual data for Fig 8 (Relative Fluorescence Intensity for Rh 123). (PZF)

S9 Data Individual data for Fig 10 (Percentage of Positive Cells for DCFH-DA). (PZF)

S10 Data Individual data for Fig 10 (Relative Fluorescence Intensity for DCFH-DA). (PZF)

S11 Data Individual data for Fig 11. (PZF)

S12 Data Individual data for Fig 12. (PZF)

S13 Data Individual data for Fig 13. (PZF)

S14 Data Individual data for Fig 14. (PZF)

S1 File The Animal Research Reporting In Vivo Experiments (ARRIVE) Guidelines Checklists. (PDF)
